# LncRNA LINC00668 promotes cell proliferation, migration, invasion ability and EMT process in hepatocellular carcinoma by targeting miR-532-5p/YY1 axis

**DOI:** 10.1042/BSR20192697

**Published:** 2020-05-11

**Authors:** Wei Xuan, Chen Zhou, Guangqiang You

**Affiliations:** 1 Department of Hepatobiliary-Pancreatic Surgery, the Third Hospital of Jilin University, Changchun, 130031, Jilin, China; 2 Personnel Department, the First Affiliated Hospital of Jilin University, Changchun, 130031, Jilin, China; 3 Department of Hepatobiliary and Pancreatic Surgery, the Second Affiliated Hospital of Jilin University, No. 218 Ziqiang Street, Nanguan District, Changchun 130031, Jilin, China

**Keywords:** HCC, LINC00668, Long non-coding RNAs, miR-532-5p, Tumorigenesis, YY1

## Abstract

Liver cancer is now one of the most lethal and commonest cancers in the world, among which over 90% is hepatocellular carcinoma (HCC). Recent studies have confirmed long non-coding RNAs (lncRNAs) are implicated in carcinogenesis. It has been reported lncRNA LINC00668 serves as an oncogene in several cancers. However, the mechanism where LINC00668 regulates HCC is still unclear. qRT-PCR analysis was adopted to detect the expression of relative RNAs. Cytoplasmic and nuclear RNA fraction analysis was conducted to verify the underlying molecular mechanism. Cell colony formation was carried out to test cell colony formation ability and transwell assays were performed to testify cell migratory and invaded abilities. Relevant protein expression level was measured by Western blot assay. LINC00668 was significantly up-regulated in HCC tissues and cell lines. LINC00668 knockdown inhibited cell proliferative, migratory and invasion abilities and slowed down the epithelial–mesenchymal transition (EMT) process. Mechanistically, LINC00668 positively modulates the expression of YY1 by competitively binding to miR-532-5p. It was revealed that LINC00668 up-regulation accelerated cell proliferation and motility in HCC and suggested LINC00668 could be a potential therapeutic target for HCC.

## Introduction

Hepatocellular carcinoma (HCC), the most prevalent subtype of liver cancer, with a steadily rising incidence, remains the fifth most common cancer worldwide [1,2]. Various therapeutic strategies have been tried for different stages of HCC patients, yet the prognosis outcomes donot seem optimistic [3]. Thus an efficient method for treatment of HCC is urgent.

Only 2% of human genome is protein-coding genes while 98% is non-coding genes [4], among which, those with at least 200 nucleotides in length are defined as long non-coding RNAs (lncRNAs) [5]. Increasing evidence has proved lncRNAs play a rather crucial role in pathophysiological process including tumorigenesis [6–10]. LncRNAs regulate biological behaviors including cell proliferation, cell apoptosis, cell metastasis etc., during tumorigenesis [11–13]. For instance, lncRNA NORAD promoted cell proliferation by competitively binding with miR-155-5p [14]. LINC00339 accelerated cell metastasis in pancreatic cancer by sponging miR-497-5p and modulating IGF1R [15]. FOXO1 suppressed cell metastasis by down-regulating AKT and inhibited lung cancer [16]. Hence, targeting lncRNAs could be considered as another approach to prevent and treat HCC [17–20]. According to previous study, it has been proved that LINC00668 is an oncogene in several cancers such as non-small-cell lung cancer [21] and gastric cancer [22]. Furthermore, LINC00668 was found prominently elevated in tumor tissue samples and cancerous cell lines. However, the functions and mechanisms of LINC00668 with respect to HCC remain unexplored. Thus LINC00668 was determined to be the lncRNA candidate.

Notably, ceRNA mechanism is prevalent recently [23]. For example, LINC00668 aggravated non-small lung cancer by targeting miR-193a/KLF7 axis [24]. However, whether LINC00668 modulates HCC through ceRNA mechanism remains to be explored. The present study is aimed to discover how LINC00668 affects biological effects of HCC and whether LINC00668 binds with miRNA to modulate downstream mRNA, providing a potential biomarker and therapeutic target for prevention and treatment of HCC.

## Materials and methods

### Human tissue samples

A total of 40 diagnostic hepatocellular cancer patients who previously received treatment from May 2013 to May 2018 joined this experiment. The informed consents had been signed by all the patients. The present study has been approved by the Ethics Committee of the Second Affiliated Hospital of Jilin University. Samples were hepatocellular cancer tissues and adjacent non-tumor tissues and were frozen in liquid nitrogen at −80°C. None of the patients enrolling in the research underwent chemo- or radiation therapy.

### Cell culture

HCC cells (HepG2, SNU-387, MHCC-97H, Huh-7) and human normal liver cell (THLE-3) were purchased from Shanghai Institute of Cell Biology (Shanghai, China) and cultured continuously in RPMI-1640 (Thermo Fisher Scientific, Waltham, MA, U.S.A.) containing 10% fetal bovine serum (FBS; Thermo Fisher Scientific), 100 μg/ml streptomycin and 100 U/ml penicillin (Thermo Fisher Scientific). Cells were maintained under standard conditions (37°C, 5% CO_2_).

### Cell transfection

HepG2 or SNU-387 cells were seeded into six-well plates and cultured for 24 h. Short hairpin RNAs (shRNAs) against LINC00668 (sh-LINC00668#1/2/3) or YY1 (sh-YY1) and their negative controls (shNCs) were acquired from Genechem (Shanghai, China). The LINC00668 or YY1 overexpression plasmid (pcDNA3.1/LINC00668 or pcDNA3.1/YY1) and the empty pcDNA3.1 were constructed by KeygenBiotech Co. Ltd (Nanjing, China). The miR-532-5p mimics, miR-532-5p inhibitors and miR-NCs were purchased from GenePharma (Shanghai, China). Finally, the transfection process lasted 48 h using Lipofectamine 2000 (Invitrogen, Carlsbad, CA, U.S.A.) and cells were collected.

### qRT-PCR analysis

RNeasy Mini Kit (Qiagen, Valencia, CA, U.S.A.) was applied as per the suggestions for isolating total RNA which was reverse transcribed to cDNA by a Reverse Transcription Kit (Invitrogen). qRT-PCR was carried out under an ABI Prism7500 fast real-time PCR system (Applied Biosystems, Foster City, CA, U.S.A.) via mixing a QuantiTect SYBR Green PCR Kit (Qiagen). Relative RNA expression was analyzed with 2^−ΔΔ*C*_t_^ approach with normalization to GAPDH or U6 RNA.

### Colony formation assay

Transfected HepG2 or SNU-387 cells were inoculated into six-well culture plates to culture for approximately 2 weeks. Colonies were generated and fixed for 15 min in 4% paraformaldehyde (PFA; Sigma–Aldrich, St. Louis, MO, U.S.A.), staining for 15 min in 0.1% Crystal Violet (Sigma–Aldrich). Colonies were imaged and analyzed afterwards.

### Subcellular fractionation

For the purpose of isolating the nuclear and cytoplasmic fractions, a Nuclear/Cytosol Fractionation Kit (Biovision, San Francisco Bay, CA, U.S.A.) was adopted. Expression patterns of LINC00668, U6 and GAPDH in nuclear and cytoplasm fractions were assayed respectively with qRT-PCR.

### Western blot

RIPA lysis buffer (Beyotime, Shanghai, China) was applied to extract complete protein based on the specification of the manufacturer. Equal amounts of proteins were subjected to SDS/PAGE (Bio-Rad, Hercules, CA, U.S.A.) and then transferred to a polyvinylidene fluoride (PVDF) (Sigma–Aldrich). Then, PVDF was sealed with skin milk. The membrane was cultured with primary antibodies at 4°C overnight and antibodies were as follows: anti-E-cadherin (ab194982, Abcam, Cambridge, U.S.A.), anti-N-cadherin (ab202030, Abcam), anti-Vimentin (ab193555, Abcam), anti-YY1 (ab109237, Abcam) and anti-GAPDH (ab8245, Abcam). The proteins were visualized via Chemiluminescence detection system (GE Healthcare, Chicago, IL, U.S.A.). Besides, GAPDH was seen as a loading control.

### Transwell assay

Transfected HepG2 or SUN-387 cells (2 × 10^4^) in every group were resuspended in serum-free medium and seeded into the top chamber of each transwell, the lower chamber was filled with 10% FBS. Invasion assay was pre-coated with Matrigel (BD Biosciences, Franklin Lakes, NJ, U.S.A.) and migration assay was not. After scheduled time, cells were fixed using methanol (Serang Biotechnology, Chengdu, China) and dyed using Crystal Violet (Amresco, Solon, OH, U.S.A.). Five randomly selected fields under 200× microscope (Olympus Corp, Tokyo, Japan) were employed for calculating the number of cells.

### Luciferase reporter assays

LINC00668-WT/Mut or YY1-WT/Mut was inserted into pmirGLO dual-luciferase vector (Promega, Madison, WI, U.S.A.) to construct pmirGLO-LINC00668-WT/Mut or pmirGLO-YY1-WT/Mut. Sequentially, pmirGLO-LINC00668-WT/Mut was co-transfected into HepG2 or SNU-387 cells with miR-532-5p mimics or miR-NC. Meanwhile, miR-532-5p mimics or miR-532-5p mimics+pcDNA3.1/LINC00668 or miR-NC and pmirGLO-YY1-WT/Mut were co-transfected into HepG2 or SNU-387 cells. Upon above procedures, luciferase signals were subjected to Dual Luciferase Reporter Assay System (Promega) as per its instructions.

### RNA immunoprecipitation assay

HepG2 or SNU-387 cells were lysed in RNA immunoprecipitation (RIP) lysis buffer (Millipore, Billerica, MA, U.S.A.). After that, cell extract was cultured with RIP buffer added with magnetic beads (Invitrogen) conjugated with anti-Ago2 (Millipore) and anti-IgG (Millipore). The sample was incubated with protease K, then shaken and digested. Immunoprecipitated RNA was isolated. LINC00668, miR-532-5p and YY1 were determined via qRT-PCR.

### RNA pull-down assay

Bio-miR-532-5p and non-bio were synthesized by Thermo Fisher Scientific. The biotinylated micRNA was incubated overnight with cell lysates (Millipore), followed by adding streptaviden magnetic beads (Invitrogen). Finally, qRT-PCR was used to detect expression levels.

### *In vivo* tumorigenesis assay

Male nude mice at the age of 6 weeks were maintained in micro-isolator cages. Mice were purchased from SIPPR-BK Laboratory Animal Co. Ltd. (Shanghai, China). After approved by Ethics committee of the Second Affiliated Hospital of Jilin University, all animal work taken place in specific-pathogen free (SPF) laboratory on the basis of institutional guidelines authorized by the Use Committee for Animal Care. After being resuspended in PBS (Sigma–Aldrich) with Matrigel, 5 × 10^6^ of cells was subcutaneously inoculated into each mouse. Every 4 days, the tumors were observed. The tumors volume was calculated based on the formula: length × width2/2. Tumors weight was detected after mice were sacrificed through cervical dislocation at the end of 4 weeks. Then, tumor tissue was extracted, followed by further PCNA or Ki67 staining.

### Statistical analysis

All experiments mentioned above were required to conduct three times independently. Statistics were analyzed using GraphPad Prism 7.0 (GraphPad Software, La Jolla, CA, U.S.A.), and shown as mean values ± standard deviation (SD). Student’s *t* test or one-way analysis of variance (ANOVA) was employed to analyze the difference. *P*-value less than 0.05 indicated statistical significance.

## Results

### LINC00668 was prominently up-regulated in HCC and knockdown of LINC00668 attenuated cell proliferation, migratory and invaded abilities and epithelial–mesenchymal transition process *in vitro*

**Figure 1:**
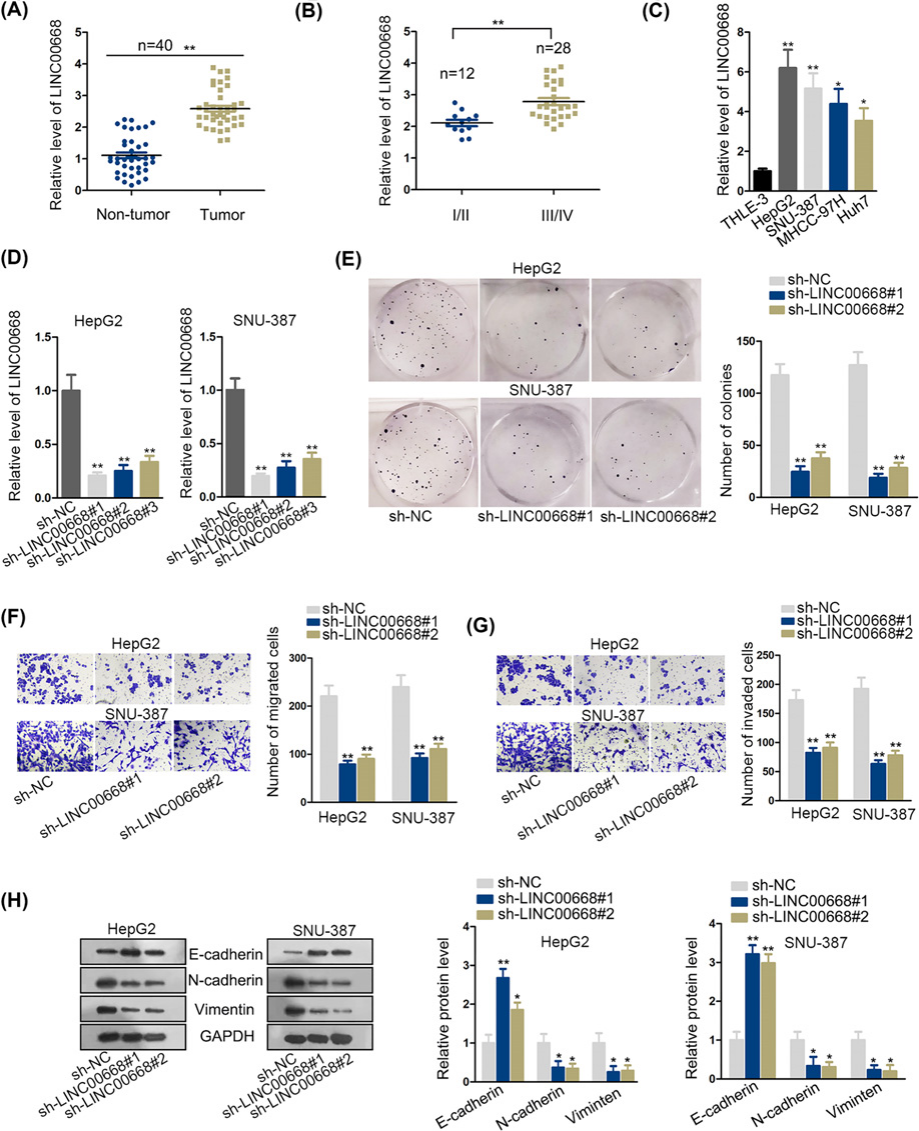
LINC00668 was prominently up-regulated in HCC and knockdown of LINC00668 attenuated cell proliferation, migratory and invaded abilities and EMT process *in vitro* (**A,B**) qRT- PCR analysis was performed to detect the LINC00668 expression level in HCC tissues the adjacent non-tumor tissues and different stages of HCC. (**C)** The expression of LINC00668 in normal liver cell line THLE-3 and HCC cell lines (HepG2, SNU-387, MHCC-97H, Huh7) was examined by qRT-PCR analysis. (**D**) The efficiency of LINC00668 knockdown was verified by qRT-PCR analysis. (**E)** Cell colony formation assay was conducted to detect cell proliferation ability after knockdown of LINC00668. (**F,G)** Transwell assays were performed to examine cell migration and invasion after knockdown of LINC00668. (**H**) EMT process changing was detected by Western blot assay after down-regulated LINC00668. *P<0.05, **P<0.01. Abbreviation: EMT, epithelial–mesenchymal transition.

To determine the expression pattern of LINC00668, we selected 40 paired tumor and adjacent normal tissue specimens. After qRT-PCR analysis, we verified that LINC00668 did present a prominent up-regulation in HCC tumor tissues (Figure 1A). Then qRT-PCR analysis also proved tumor tissues at advanced stages of HCC demonstrated a significantly higher expression of LINC00668 than those at early stages of HCC (Figure 1B). Furthermore, LINC00668 expression was found strikingly elevated in carcinoma cell lines (HepG2, SNU-387, MHCC-97H, Huh7) compared with normal liver cell line THLE-3 (Figure 1C). Therefore, we may conjecture LINC00668 plays a crucial role in HCC and might function as an oncogene in HCC progression.

For further study, we determined to examine cell biological effects after knockdown of LINC00668. Presenting prominent expression of LINC00668, HepG2 and SNU-387 were chosen as our basic research object. Next, HepG2 and SNU-387 cells were transfected with sh-LINC00668 and sh-negative control (sh-NC). LINC00668 from group sh-LINC00668#1 and sh-LINC00668#2 were confirmed to be more efficiently knocked down than sh-LINC00668#3 after qRT-PCR analysis (Figure 1D). Consistently, we examined cell colony numbers and detected attenuation of cell colony formation ability, indicating knockdown of LINC00668 efficiently suppressed proliferative ability (Figure 1E and Supplementary Figure S1). Next, transwell assays were conducted to test cell migratory and invaded abilities. According to results, migrated and invaded cell numbers significantly went down after LINC00668 depletion, suggesting impairment in cell motility ability (Figure 1F,G). Furthermore, Western blot assay was conducted to test epithelial–mesenchymal transition (EMT) process. The results presented that E-cadherin increased while N-cadherin and Vimentin decreased after knockdown of LINC00668, which suggested LINC00668 knockdown inhibited cell EMT process (Figure 1H). In a word, LINC00668 was up-regulated in HCC and LINC00668 silence retarded the process of HCC.

### LINC00668 serves as a molecular sponge for miR-532-5p in HCC cells

ceRNA mechanism, prevalent recently, refers to lncRNA competes with mRNA to bind with miRNA. More and more studies tend to explore the ceRNA mechanism in various cancers and make contribution to theoretical support for clinical treatment. Besides, as sh-LINC00668#1 presented higher inhibition efficiency in functional assays (Figure 1), we employed cells transfected with sh-LINC00668#1 (named as sh-LINC00668 subsequently) to continue the following studies.

**Figure 2:**
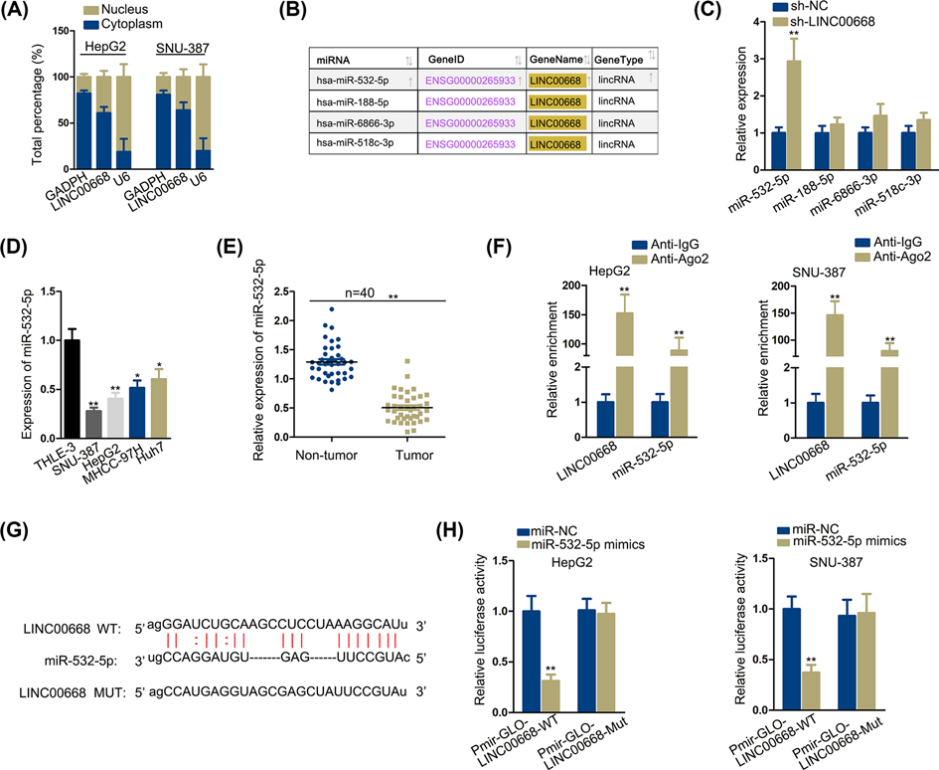
LINC00668 serves as a molecular sponge for miR-532-5p in HCC cells (**A**) Cytoplasmic and nuclear RNA fraction of HCC cells was carried out. (**B**) Screening of matched miR-532-5p from StarBase was presented. (**C**) qRT-PCR analysis presented the relative expression of the four screened microRNAs. (**D,E**) MiR-532-5p expression was tested by qRT-PCR analysis in normal liver cell line and cancerous cell lines/peritumoral tissues and tumor tissues. (**F**) RIP assay were conducted to see the expression and binding situation of LINC00668 and miR-532-5p. (**G**) The predicted binding sites between LINC00668-WT and miR-532-5p and the mutant sequence of LINC00668 were demonstrated. (**H**) Luciferase reporter assay were conducted to see the expression and binding situation of LINC00668 and miR-532-5p. *P<0.05, **P<0.01.

In our study, first we examined location of LINC00668. Using cytoplasmic and nuclear RNA fraction technology, it was testified LINC00668 was predominantly distributed in cytoplasm (Figure 2A), indicating a possibility of ceRNA mechanism. Then by retrieving StarBase V2.0 (http://starbase.sysu.edu.cn/), four microRNAs that could bind with LINC00668 were selected (Figure 2B). Among which, qRT-PCR examination detected miR-532-5p expression conspicuously rose after knockdown of LINC00668 in comparison with other three microRNA candidates (Figure 2C). Furthermore, we testified miR-532-5p expression respectively in normal liver cell line THLE-3 and four cancerous cell lines (HepG2, SNU-387, MHCC-97H, Huh7), finding miR-532-5p was up-regulated prominently in THLE-3 compared within cancerous cell lines (Figure 2D). Also, qRT-PCR examination detected elevated expression of miR-532-5p in peritumoral tissues than in tumor tissues (Figure 2E). Taken that, we may conclude that *miR-532-5p* is a tumor-suppressing gene. RIP technology was carried out next and data manifested that LINC00668 and miR-532-5pwere enriched in Ago2 antibody, further confirming the ceRNA mechanism (Figure 2F). Furthermore, we performed luciferase reporter assay. StarBase V2.0 have given a predicted binding sites between LINC00668 and miR-532-5p (Figure 2G), based on which, we mutated the binding sites of LINC00668 and respectively constructed them on luciferase reporter gene vector. Data revealed that miR-532-5p up-regulation made a decline in luciferase activity of pmirGLO-LINC00668-WT but failed to affect luciferase activity of pmirGLO-LINC00668-Mut (Figure 2H). To sum up, LINC00668 could bind to miR-532-5p in HCC cells.

### LINC00668 exerts positive influence on YY1 by sponging miR-532-5p

**Figure 3:**
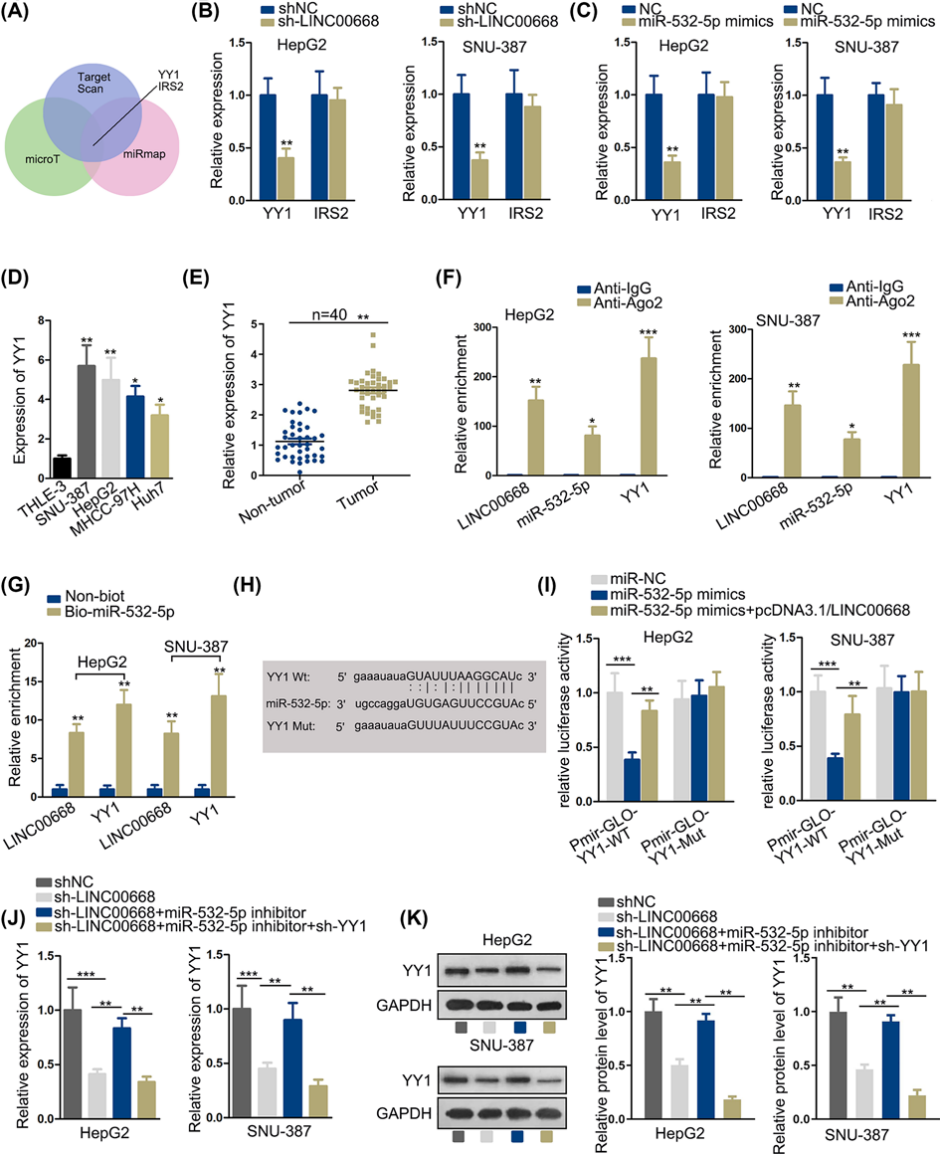
LINC00668 exerts positive influence on YY1 by sponging miR-532-5p (**A**) Qualified mRNA candidates were selected. (**B,C**) mRNA candidates’ expression were examined by qRT-PCR analysis after knockdown of LINC00668 and overexpression of miR-532-5p. (**D,E**) qRT-PCR analysis presented the YY1 expression in normal liver cell line/cancerous cell lines and peritumoral tissues and tumorous tissues. (**F**–**I**) RIP assay, pull-down assay and luciferase reporter assay were performed to verify the relationship among LINC00668, miR-532-5p and YY1. (H) The predicted binding sites between YY1-WT and miR-532-5p and the mutant sequence of YY1 were demonstrated. (**J**) The expression of YY1 and according protein level in HepG2 and SNU-387 cells after intervene of LINC00668, miR-532-5p and YY1 were detected by qRT-PCR. (**K**) The YY1 protein level was detected by Western blot. *P<0.05, **P<0.01, ***P<0.001.

By means of StarBase, we screened qualified mRNA by designating relevant data (clade: mammal; genome: human; assembly: hg19; miRNA: miR-532-5p; CLIP data: medium stringency; degradome data: medium stringency; pan-cancer ≥ 6; program number: 3; predicted program: microT, miRmap, TargetScan). mRNA YY1 and IRS2 were selected after screening (Figure 3A). After knockdown of LINC00668 and overexpression of miR-532-5p in HepG2 and SNU-387, YY1 was observed with an obvious rise compared with normal control while no overt change could be seen in IRS2 expression, determining YY1 to be the downstream target gene of miR-532-5p (Figure 3B,C). Furthermore, YY1 expression was prominently elevated in cancerous cell lines and tumor tissues than in normal cell line and peritumoral tissues by qRT-PCR analysis (Figure 3D,E). Then, we analyzed the relationship among LINC00668, YY1 and miR-532-5p. RIP assay was carried out next and enrichment level of RNAs was examined by qRT-PCR analysis. Based on the results of RIP assay, we found LINC00668, miR-532-5p and YY1 were enriched in Ago2 groups but rare in IgG groups, verifying LINC00668, YY1 and miR-532-5p coexisted in RNA-induced silencing complexes (RISCs) (Figure 3F). Furthermore, RNA pull-down assay was conducted to evaluate the binding situation among LINC00668, miR-532-5p and YY1. According to the results, LINC00668 and YY1 expression was detected, confirming LINC00668 and YY1 were pulled down by biotinylated miR-532-5p (Figure 3G). By means of StarBase V2.0, we found predicted binding sites between YY1 and miR-532-5p and we created a mutant type of YY1 to verify the veracity of binding sites (Figure 3H). Next, luciferase reporter assay was conducted to further confirm the relationship among three objects. The results demonstrated cotransfection of the luciferase reporter plasmids containing YY1-WT and miR-532-5p mimics into HepG2 and SNU-387 cells led to decrease in luciferase activity, after transfection of pcDNA3.1/LINC00668, the luciferase activity recovered to some extent, while up-regulation of miR-532-3p and overexpression of LINC00668 had no effects on the luciferase activity of plasmids contained YY1-Mut (Figure 3I). In addition, we testified the expression of YY1 after LINC00668 intervened, miR-532-5p and YY1, finding a negative regulation relationship between YY1and miR-532-5p and a positive regulation relationship between LINC00668 and YY1 (Figure 3J). The following YY1 protein level test disclosed an according change with YY1 mRNA expression (Figure 3K). In summary, LINC00668 exerted positive effects on YY1 via sponging miR-532-5p.

### LINC00668 accelerates cell proliferation, migratory and invasion abilities and EMT process by targeting miR-532-5p/YY1 axis

**Figure 4:**
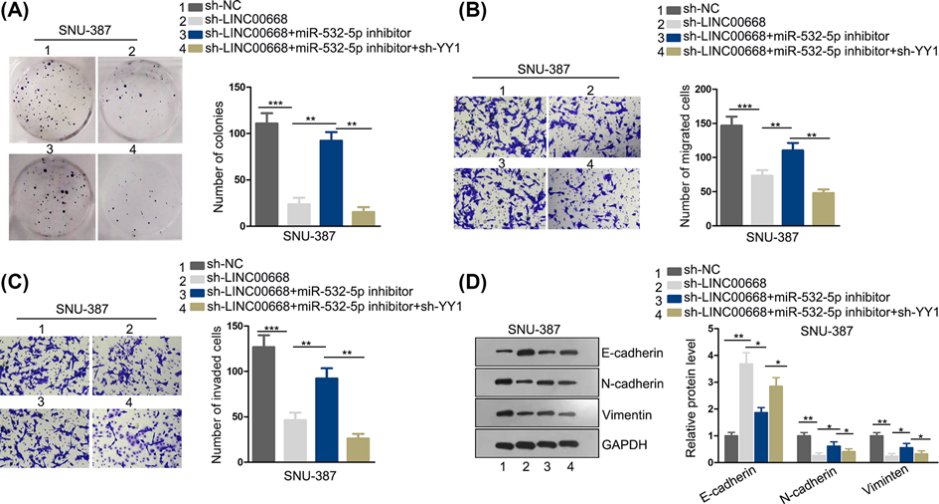
LINC00668 accelerates cell proliferation, migratory and invasion abilities and EMT process by targeting miR-532-5p/YY1 axis (**A**) Cell proliferation ability was examined by cell colony formation assay after intervene of LINC00668, miR-532-5p and YY1. (**B,C**) Transwell assays were performed to detect cell migration and invasion ability after intervention of LINC00668, miR-532-5p and YY1. (**D**) EMT process was detected using Western blot assay after intervene of LINC00668, miR-532-5p and YY1. *P<0.05, **P<0.01, ***P<0.001.

To further confirm whether LINC00668 promotes HCC by targeting miR-532-5p/YY1 axis and accordingly modulates biological behaviors, we have carried out a series of function rescue experiments. First, cell colony formation assay was conducted. Cell colony formation ability decreased after sh-LINC00668 plasmids were transfected into SNU-387 cell, while this falling trend was recovered after down-regulation of miR-532-5p but then decreased again after knockdown of YY1 (Figure 4A). In addition, transwell assays were performed and cell migration along with invasion decreased after knockdown of LINC00668, restored after depletion of miR-532-5p while lessened again after YY1 silence (Figure 4B,C). Furthermore, EMT process was slowed down due to knockdown of LINC00668, whereas such phenomenon was rescued when miR-532-5p was down-regulated and diminished again caused by knockdown of YY1 (Figure 4D). Collectively, above results indicate LINC00668 accelerated cell proliferation, migration and invasion by sponging miR-532-5p and modulating YY1.

### The effects imposed via LINC00668 silence on tumor growth *in vivo* could be reversed by YYI up-regulation

**Figure 5:**
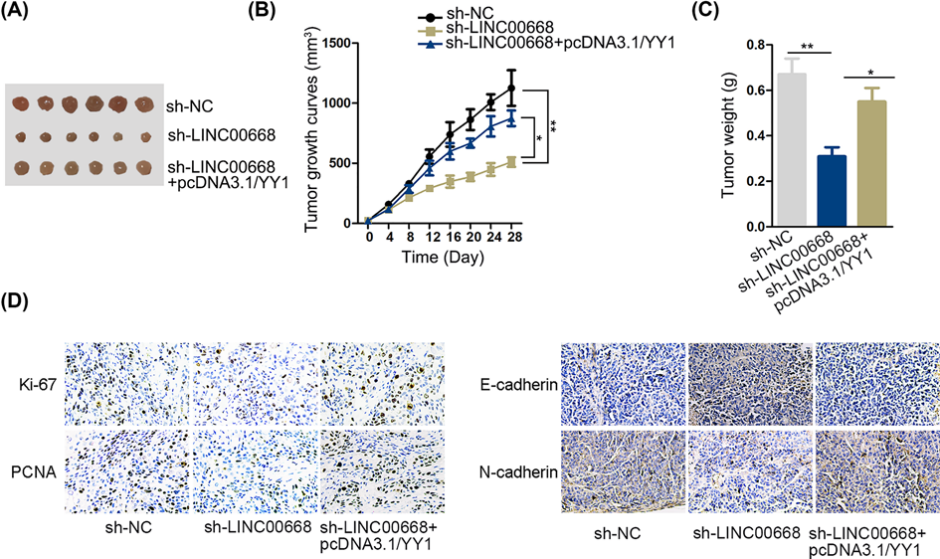
The effects imposed via LINC00668 silence on tumor growth *in vivo* could be reversed by YYI up-regulation (**A**) Pictures of tumors in sh-NC, sh-LINC00668 and sh-LINC00668+pcDNA3.1/YY1 group. (**B**) Tumor growth curve in different groups**.** (**C**) Tumor weight in different groups. (**D**) IHC examined Ki67, PCHA, E-cadherin and N-cadherin in different groups. *P<0.05, **P<0.01.

Afterward, to validate LINC00668 promoted tumor growth through modulating YY1 expression further, we conducted *in vivo* experiments. We classified all nude mice into three groups and separately injected the cells transfected with sh-NC, sh-LINC00668 and sh-LINC00668+pcDNA3.1/YY1. The volume was measured every 4 days. At the end of 28th day, all mice were killed and took out the tumors. They were shown in Figure 5A. According to the volume growth curve in Figure 5B, LINC00668 knockdown overtly suppressed the growth of tumor. Meantime, up-regulation of YY1 restored the impacts of LINC00668 silencing. Additionally, the same results could be discovered in tumor weight (Figure 5C). The outcomes of IHC revealed that up-regulation of YY1 reversed the effects of down-regulated LINC00668 on the expression E-cadherin, N-cadherin, Ki67 and PCNA as well (Figure 5D). Altogether, LINC00668 silence could be reversed by overexpression of YYI *in vivo* experiments.

## Discussion

More and more cancer-related lncRNAs have been identified recently. Many lncRNAs were proved to have effects on HCC progression, among which, LINC00668 was found to serve as an oncogene in several cancers such as colorectal cancer [25] and lung adenocarcinoma [26]. LncRNA LINC00668 was reported to accelerate progression of breast cancer [27]. Besides, it pointed that LINC00668 suppressed apoptosis rate and facilitated cell cycle in breast cancer. These findings revealed that LINC00668 was an oncogene in breast cancer. However, relevant research on functions and mechanisms by which LINC00668 modulates HCC has not been carried out. In our study, first, we detected LINC00668 expression respectively in non-tumor tissues and tumor tissues and different stages of HCC. The results indicate that LINC00668 was prominently elevated in tumor tissues and advanced stage of HCC. Besides, here we discovered high expression of LINC00668 was in HCC cell lines compared with normal. However, LINC00668 expression in different HCC cell lines were inequitable, and this might be attributed to the malignancies of these cell lines since SNU-387 cells were from primary hepatic carcinoma), MHCC-97H cells were from high metastatic liver cancer, HepG2 cells were extracted from liver cancer tissue in a 15-year-old white teenage and Huh7 cells were highly differentiated HCC cells which has low malignancy. Though MHCC-97H from highly metastatic tumors should be more malignant, LINC00668 expression in this cell line was not particularly high, which may be caused by tumor heterogeneity to some extent. Furthermore, knockdown of LINC00668 suppressed cell proliferation, migration along with invasion and EMT process, suggesting an oncogenic role of LINC00668 and the expression of LINC00668 is positively associated with advanced TNM stage in HCC.

Increasing evidence suggests lncRNA is a natural miRNA sponge and regulates biological functions. For example, lncRNA 00707 promotes colorectal cancer by binding with miR-206 [28]. SNHG6 aggravates HCC by competitively binding to miR-139-5p [29]. To explore the underlying molecular mechanism LINC00668 applied for, we conducted cytoplasmic and nuclear RNA fraction analysis and found LINC00668 was mostly distributed in cytoplasmic RNA, which indicated the possibility of ceRNA mechanism. Then, by screening from StarBase V2.0, we discovered several miRNAs that share binding sites with LINC00668. After knockdown of LINC00668, miR-532-5p expression was prominently elevated compared with the other three miRNAs. Furthermore, miR-532-5p expression is also found up-regulated in tumor adjacent normal tissues and normal liver cell line than in tumor tissues and cancerous cell lines. According to previous study, miR-532-5p was as well verified to serve as tumor suppressor in epithelial ovarian cancer [30]. In the recent study, miR-532-5p was discovered to have inhibitory function in glioma cells via targeting CSF1 [31]. Based on this finding and the expression of miR-532-5p in HCC tissues and cells, we inferred miR-532-5p was a tumor suppressor in HCC as well. After verification of RIP assay and luciferase reporter assay, we confirmed miR-532-5p to be the downstream RNA of LINC00668.

Also, we identified mRNA YY1 using StarBase V2.0 as the target of miR-532-5p. It has been reported that YY1 functions as an oncogene in triple negative breast cancer [32]. Thus we may conjecture YY1 as an oncogene in HCC. Lately, YY1 was found as a novel target for diabetic nephropathy orchestrated renal fibrosis [33], suggesting decreasing YY1 expression was beneficial for diabetic nephropathy treatment, which offered us insight for diminish YY1 expression in HCC cells. Furthermore, according to the results, the expression of YY1 in tumor tissues was up-regulated significantly than that in non-tumor tissues. A positive correlation between LINC00668 and YY1 and a negative correlation between miR-532-5p and YY1 was observed in HCC tissues. Furthermore, knockdown of YY1 reversed the function of miR-532-5p and recovered cell proliferation, migratory and invasion capacities. More importantly, we used mice experiments to certify LINC00668 silencing could hamper the growth of HCC and up-regulation of YY1 could countervail the influence induced by LINC00668 knockdown.

Therefore, we may conclude that LINC00668 sponges miR-532-5p to modulate YY1 expression in HCC. In other words, LINC00668 promotes HCC tumorigenesis by binding with miR-532-5p thus modulating YY1. Taken that, LINC00668- miR-532-5p - YY1 mechanism might hopefully provide some theoretic basis for HCC diagnosis and treatment.

## Supplementary Material

Supplementary Figure S1

## Abbreviations

Ago2: argonaute 2
cDNA: complementary DNA
ceRNA: competing endogenous RNA
GAPDH: glyceraldehyde-3-phosphate dehydrogenase
HCC: hepatocellular carcinoma
IgG: immunoglobulin G
IRS2: insulin receptor substrate 2
Ki67: proliferation marker protein Ki-67
LINC00668: long intergenic non-coding RNA No. 668
miRNA: microRNA
NC: negative control
NORAD: non-coding RNA activated by DNA damage
PCNA: proliferating cell nuclear antigen
qRT-PCR: quantitative real time polymerase chain reaction
RPMI-1640: Roswell Park Memorial Institute No. 1640
SNHG6: small nucleolar RNA host gene 6
TNM: tumor node metastasis
YY1: Yin Yang-1
