# Supplementary material for: LncRNA LINC00668 promotes cell proliferation, migration, invasion ability and EMT process in hepatocellular carcinoma by targeting miR-532-5p/YY1 axis
Source: Biosci Rep. 2020 May 11;40(5):BSR20192697. doi: 10.1042/BSR20192697 (PMC7214398; doi:10.1042/BSR20192697)
Supplement: Supplementary Figure S1 [file BSR-2019-2697_supp.pdf]

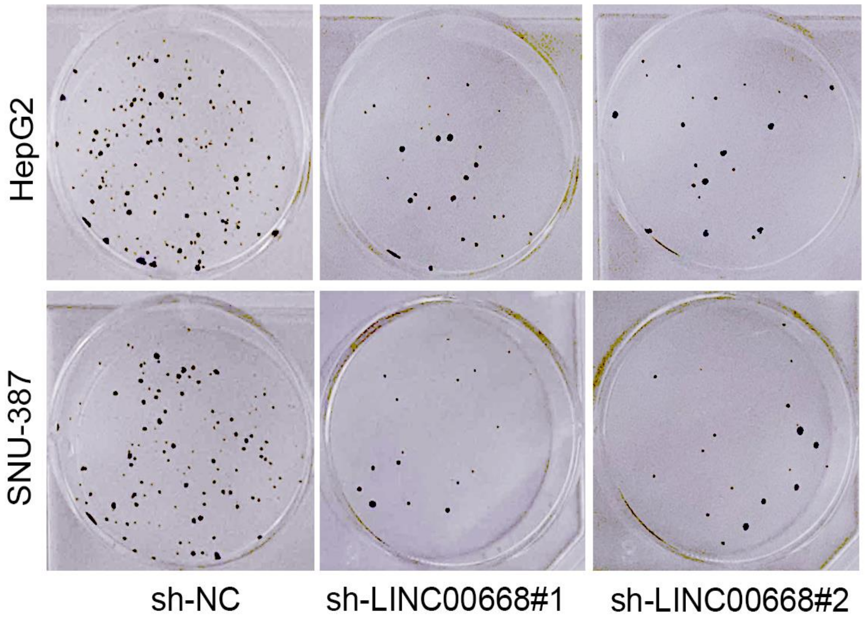
HepG2
SNU-387
sh-NC
sh-LINC00668#1
sh-LINC00668#2

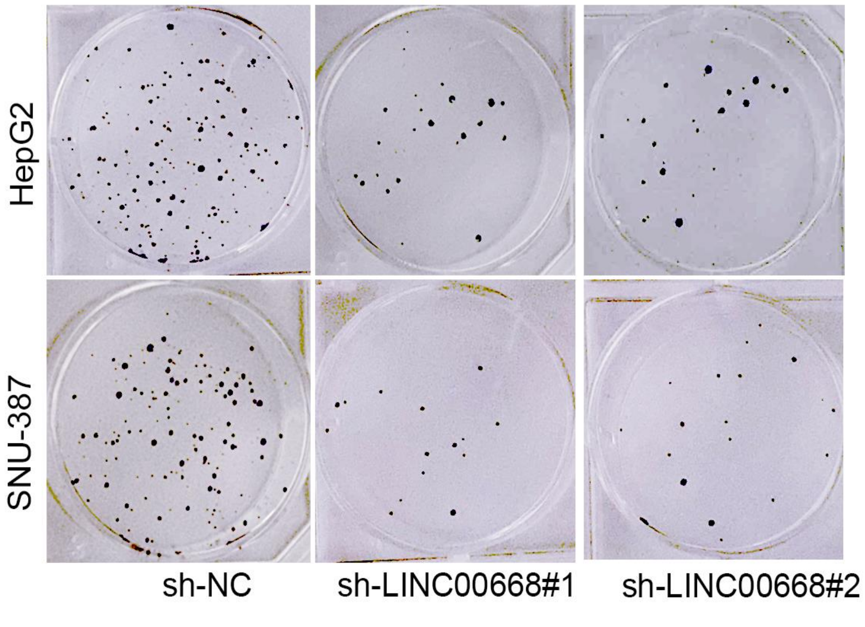
HepG2
SNU-387
sh-NC
sh-LINC00668#1
sh-LINC00668#2

Supplementary Figure 1 Pictures of another twoindependent colony formation experiments conducted in cells facing knockdown of LINC00668.
